# Photoprotective strategies in pale versus melanic boreal hair lichens: non-photochemical quenching compensates for less protective fungal pigments

**DOI:** 10.1007/s00425-025-04720-2

**Published:** 2025-05-22

**Authors:** Knut Asbjørn Solhaug, Yngvar Gauslaa

**Affiliations:** Faculty of Environmental Sciences and Natural Resource Management, Norwegian University of Life Sciences, P. O. Box 5003, NO-1432 Ås, Norway

**Keywords:** *Alectoria*, *Bryoria*, Boreal forest, Light stress adaptation, Melanin, Usnic acid, Non-photochemical quenching

## Abstract

**Main conclusion:**

Hair lichen photoprotection involves algal and fungal strategies while hydrated, with pale lichens compensating weak fungal cortical pigments through high, rapidly induced non-photochemical quenching by the photobiont.

**Abstract:**

Hair lichens play vital roles in boreal forests by influencing nutrient cycles, microclimates, and providing habitats for invertebrates and forage for animals. This study examines two widespread and dominant species, *Bryoria fuscescens* and *Alectoria sarmentosa*, which possess different fungal pigments—dark light-absorbing melanin in *Bryoria*, and pale reflecting usnic acid in *Alectoria*. These cortical pigments affect species distribution, with *Bryoria* favoring sun-exposed forest canopies due to its efficient light-protective melanin, while *Alectoria* thrives in partly shaded, moist environments. By investigating sympatric populations, we explored whether non-photochemical quenching (NPQ) compensates for *Alectoria*’s less-effective sun-screening pigment. Our results reveal that *Alectoria* exhibits higher and more rapidly induced NPQ compared to *Bryoria*, along with faster recovery from photoinhibition. The flexibility and rapid response of *Alectoria*’s NPQ help mitigate high-light stress, optimizing growth in fluctuating light environments. These compensatory mechanisms suggest that, despite weaker cortical pigmentation, hydrated *Alectoria* can sustain photosynthesis and recover from light-induced damage more efficiently. However, because NPQ does not function in the desiccated state—where efficient sun-screening by cortical pigments is essential—*Alectoria* is confined to humid and sheltered forest canopies in drier macroclimates but not in rainforest climates. This study underscores the adaptive strategies of both photobionts and mycobionts in hydrated hair lichens to manage varying light conditions in boreal forests, highlighting NPQ as a compensating mechanism in lichen photoprotection. It advances our understanding by illustrating how the transition from dry to wet conditions amplifies the algal partner’s contribution to overall photoprotection.

## Introduction

Thin hair lichens are often dominant in boreal forest canopies (Ahti 1977), forming a crucial component of these ecosystems (Edwards et al. 1960; Campbell and Coxson 2001; Stevenson 2001). They substantially increase in biomass with stand age (Esseen et al. 1996; Price and Hochachka 2001; Boudreault et al. 2015), trapping nutrients and water, and influencing canopy microclimate (Knops et al. 1996; Pypker et al. 2017). Additionally, hair lichens provide essential habitat for invertebrates, which serve as prey for various birds (Pettersson et al. 1995), and are a vital winter forage for animals such as reindeer (Heggberget et al. 2002; Horstkotte et al. 2011) and the threatened mountain caribou (Rominger and Oldemeyer 1990; Rominger et al. 1996; Goward et al. 2024).

The two most important hair lichen genera in boreal forest are *Alectoria* and *Bryoria*. Despite their close taxonomic relation and morphological similarities, these genera are easily distinguished by their cortical fungal pigments: the pale yellowish usnic acid in *Alectoria* and the brown melanin in *Bryoria* (Brodo and Hawksworth 1977). This study focusses on the two common and widespread species, *Alectoria sarmentosa* (Ach.) Ach. and *Bryoria fuscescens* (Gyelnik) Brodo & D. Hawksw., hereafter referred to by their genus names. Although they have similar global distributions (https://www.gbif.org/species; accessed 11 April 2025), they often occupy different niches, resulting in distinct small-scale distribution patterns (Gauslaa and Goward 2023). *Bryoria* tends to dominate exposed upper canopies and open stands near hilltops (Goward et al. 2022), whereas *Alectoria* prefers lower, shaded canopies (Benson and Coxson 2002; Coxson and Coyle 2003).

The success of melanic hair lichens in sun-exposed sites has been attributed to the efficient high light protection provided by melanin, which reduces photoinhibitory damage (Färber et al. 2014; Gauslaa and Goward 2023). Effective solar screening for susceptible photobionts is particularly crucial during sunny and dry weather, as these conditions inactivates protective physiological processes and prevents the repair of high-light damage accumulating over time (Färber et al. 2014). Thus, the photoprotective role of melanin is well established. However, it remains unclear how usnic hair lichens cope with high light and how hydration status influences photoprotection in such thin lichens.

In hydrated lichens, fungal pigments influence various functions. For instance, dark pigmentation increases the compensation point for net photosynthesis in *Bryoria* (Coxson and Coyle 2003), which may explain its lower growth rate compared to *Alectoria* in shaded canopies (Esseen and Coxson 2024). Hydrated lichens have more tools to deal with high light stress than desiccated ones. Like other photosynthetic active organisms (Jung and Niyogi 2006), hydrated photobionts can dissipate excess absorbed light safely via mechanisms such as non-photochemical quenching (NPQ; Goss and Lepetit 2015), or detoxify reactive oxygen species to avoid damage (Beckett et al. 2021). These algal photoprotective mechanisms respond much faster to changing light than the slow fungal synthesis of light-screening pigments (as discussed by Solhaug et al. 2024). Additionally, *Bryoria* retains an unusually large amount of external water (Esseen et al. 2017) compared to usnic hair lichens (Eriksson et al. 2018), likely due to the high water-binding capacity of melanin (Beilinson et al. 2022). Excess external water significantly hinders CO_2_ uptake by blocking diffusion pathways (e.g., Lange et al. 2001), which may contribute to the low growth rates of *Bryoria* in wet climates (Phinney et al. 2021). This characteristic helps explain why melanic hair lichens prefer open forest and well-ventilated canopies, where moisture levels are lower and CO_2_ diffusion is less impeded (Goward 1998; Goward et al. 2022).

Recent findings suggest that high and flexible NPQ can compensate for weak cortical pigmentation in thick mat-forming lichens that dominate alpine vegetation (Solhaug et al. 2024). Since NPQ is induced within minutes in hydrated lichens, it efficiently dissipates excess fluctuating light, optimizing lichen growth and photosynthesis during moist periods in tree canopies. Lower canopies experience temporal sunflecks (Coxson and Stevenson 2007; Way and Pearcy 2012; Demmig-Adams et al. 2014), where rapidly induced photoprotection would be particularly beneficial (Mkhize et al. 2022).

This study aims to quantify the NPQ in sympatric populations of two ecologically important and dominant hair lichens: the usnic *Alectoria* and the melanic *Bryoria*. Due to the difficulty of directly quantifying cortical light transmittance in such thin and brittle lichens, we will employ various photobiological approaches to seek indirect evidence of screening efficiency in the studied populations. The collected data will then be used to test our main hypothesis that high NPQ in hydrated hair lichens can compensate for less-efficient sun-screening pigments. Furthermore, we will provide reflectance spectra of both wet and dry hair lichens and use information from literature on high light tolerance of desiccated thalli of usnic and melanic hair lichens to establish a better understanding of why reported small-scale distribution patterns of *Alectoria* and *Bryoria* (Benson and Coxson 2002; Antoine and McCune 2004) differ between dry and humid forests.

## Materials and methods

### Lichen material

**Fig. 1:**
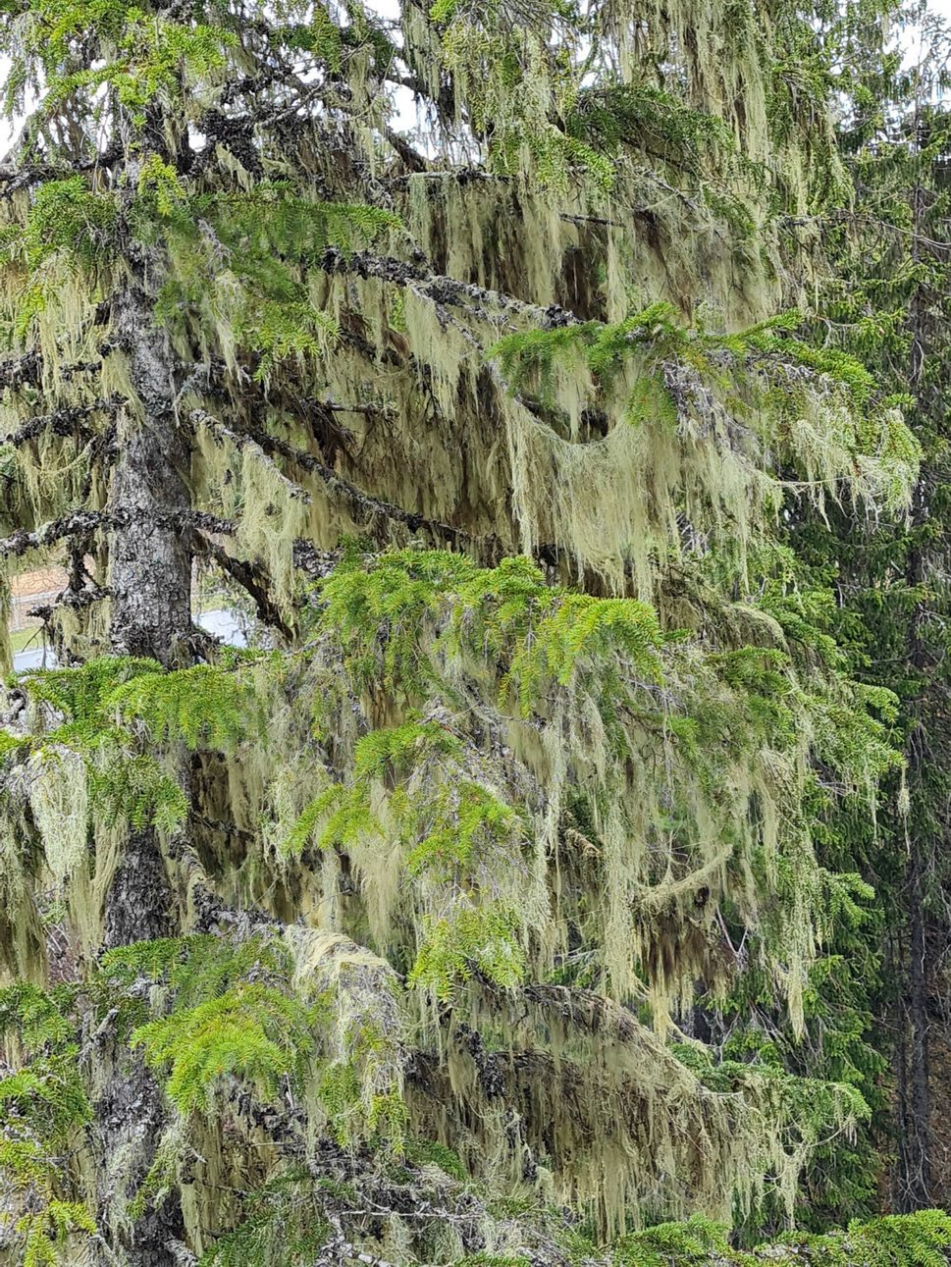
One of the *Picea abies* canopies used for sampling of hair lichen thalli in Sandvika, Verdal, Trøndelag (63.6509 N, 12.2506E, 441 m a.s.l.; annual precipitation: > 1000 mm precipitation). Note that the usnic hair lichen *Alectoria sarmentosa* dominate this rather exposed canopy, but scattered melanic *Bryoria fuscescens* thalli can be seen in between the usnic hair lichen

*Alectoria sarmentosa* and *B. fuscescens* were collected on May 24, 2023, from mixed populations on multiple trees in an open and low *Picea abies* forest (Fig. 1) situated on a northeast-facing slope in Sandvika, Verdal, Trøndelag (63.6509 N, 12.2506E, 441 m a.s.l.) in a humid, suboceanic climate with > 1000 mm precipitation a year (Moen 1999). However, the collection happened to take place at the end of an unusually dry period. The last 20 days before collection saw only minimal rain on two occasions, and April had only 45% of normal rainfall.

The lichens were dried at room temperature in the lab and then stored in a freezer (− 20 °C) until measurements were taken in October 2023, following the recommended storage protocol for later physiological studies (Honegger 2003). For each measurement specified below, new thalli were randomly selected from the freezer. They were then sprayed to saturation with deionized water and pretreated in the hydrated state for 20–24 h on moist filter paper under low light (10 µmol photons m^−2^ s^−1^) in a temperature-controlled room at 15 °C. This pre-treatment efficiently reduces the previous levels of photoinhibition (Solhaug 2018).

### Spectral reflectance

The reflectance spectra of six thalli of each species were measured on (1) air-dry and (2) fully hydrated thalli using an integrating sphere (RT Sphere, Spectral Evolution, Haverhill, MA, USA) connected to a spectrophotometer (Model RS-3500, Spectral Evolution). Each thallus was arranged to fully cover the entire measurement area of approximately 3 cm^2^ for each measurement.

### Electron transport rate (ETR)

The ETR is calculated as *Φ*_PSII_ × PAR × 0.5 × Abs (Baker 2008). *Φ*_*PSII*_ is the effective quantum yield of photosystem II (PSII); 0.5 assumes equal absorption of photons in PSII and PSI, and Abs is the fraction of incident light absorbed in PSII and PSI. *Φ*_PSII_ was measured from 0 to 803 μmol photons m^−2^ s^−1^, using a red-light imaging-PAM M-series fluorometer (Heinz Walz GmbH, Effeltrich, Germany). The Abs parameter is typically assumed to be 0.85 in green leaves, but is lower in lichens due to screening by cortical pigments (Solhaug et al. 2010). We assessed apparent ETR (ETR_App_) in 12 thalli of each species by setting Abs = 1. Because ETR_App_ does not include the unknown Abs parameter, it is higher than the real ETR. A higher ETR_App_ in one species normally implies higher cortical screening.

### Photoinhibition

Twelve hydrated thalli from each species were kept continuously hydrated during a 20-h pre-treatment at low light (10 µmol photons m^−2^ s^−1^). Afterward, all thalli were randomly placed under a LED lamp (Model SL3500, Photon System Instruments, Brno, Czech Republic) producing 750 μmol photons m^−2^ s^−1^ with equal amounts of red, green, and blue light. The lichens (checked for uniform light) were repeatedly sprayed to keep them moist during the 4 h light exposure. The pre-treatment and subsequent high-light treatment were conducted in a temperature-controlled room at 15 °C. After exposure to low light (8 µmol photons m^−2^ s^−1^) for 5 min, 30 min, 1 h, 2 h, 4 h, and 24 h (each followed by 5 min of darkness), the maximum quantum yield of PSII (*F*_*V*_*/F*_*M*_) was measured using a red LED Imaging-PAM M-series chlorophyll fluorometer and ImagingWin v2.46i software (Heinz Walz) to document the recovery kinetics after the high-light treatment.

### Non-photochemical quenching (NPQ)

New thalli, pretreated for 24 h at 10 µmol photons m^−2^ s^−1^, were used for NPQ measurements at 233 and 613 μmol photons m^−2^ s^−1^ (*n* = 12 per each species at each light level). The highest light level was chosen, because it was needed for light saturation of ETR in both species and is thus ecologically relevant. The lichens were then dark adapted for 10 min and placed in the Imaging-PAM for NPQ analyses. *F*_*M*_ was measured with a strong light flash and no actinic light, giving the fluorescence of a closed PSII. The actinic light was then turned on, and the program initiated saturating light pulses (3000 μmol photons m^−2^ s^−1^) nine times at regular intervals for 23 min. At each point, the fluorescence was measured. This was followed by nine measurements of fluorescence in the dark for 10 min. Non-photochemical quenching was calculated as NPQ = (*F*_*M*_* – F*_*M*_′)/*F*_*M*_′ where *F*_*M*_ is *F*_*M*_’ from the first measurement at PAR = 0 (Schreiber et al. 1986).

### Chlorophyll content

Chlorophyll (Chl) content was measured in the thalli used for the reflectance measurements. Approximately 15–20 mg dry, intact lichen material was weighed and placed in Eppendorf tubes. Each tube was then filled by 1.5 ml DMSO to extract the Chl over a period of 20 h in darkness at room temperature. After centrifugation, 1 ml of each solution was analyzed using a UV-2101PC spectrometer (Shimazdu Scientific Instruments, Kyoto, Japan).

The *Bryoria* extracts contained substantial amounts of melanic compounds that absorb at the wavelengths used for Chl quantification. Therefore, it was essential to separate and remove melanin using a C18 solid-phase extraction column (Agilent Bond Elut C18; Agilent Technologies Inc, Santa Clara, CA, USA). First, the raw spectrum of the DMSO extract was measured. Then, the melanin spectrum from the C18 was subtracted from the raw spectrum before computing Chl using the formulae provided by Welburn (1994). Details on the C18 solid-phase are given by Solhaug and Gauslaa (2025). No such treatment was needed for *Alectoria*.

### Statistical methods

Means and standard errors were calculated and presented in tables and figures and expressed as mean ± standard error (SE) in the text. Regression equations, along with associated 95% confidence intervals, *R*^2^_adj_, and *P* values were generated using SigmaPlot version 14.0. A Student’s *t* test was conducted to examine species-wise differences in Chl concentration and the Chl *a/b*-ratio.

## Results

### Cortical pigments shape hair lichen reflectance spectra

**Fig. 2:**
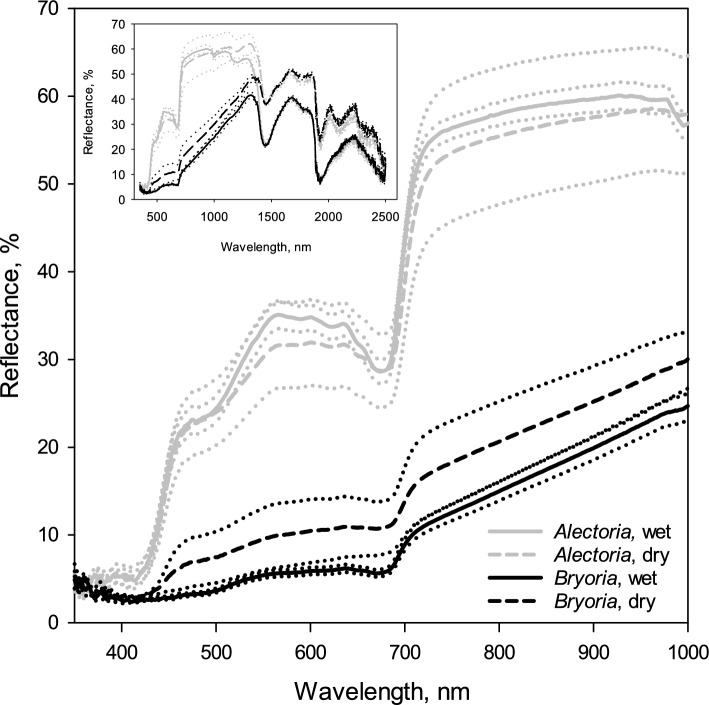
Mean reflectance spectra (350–1000 nm) from densely packed thalli of the pale usnic *Alectoria sarmentosa* and the darkly melanic *Bryoria fuscescens*. Reflectance spectra are shown for both air-dry (hatched lines) and fully hydrated thalli (solid lines) of both species The dotted lines on both sides of solid and hatched lines (mean values) show ± SE (*n* = 6). The inset shows the entire reflectance spectra of the same species from 350 to 2500 nm)

While hydrated, the reflectance of photosynthetic active light (PAR) in *Bryoria* was consistently low, never exceeding 6%. In contrast, *Alectoria* exhibited substantially higher PAR reflectance, peaking at 36% in the green part of spectrum (563–570 nm; Fig. 2). *Bryoria* displayed a subtle peak in the PAR-range at red light (632–644 nm). Both species showed a minimum in reflectance at 670–680 nm, with *Alectoria* having a much sharper minimum. From this red minimum, both species experienced a sudden rise in reflectance in the near infrared range. The rise was much stronger in *Alectoria*, which had significantly higher reflectance in the 700–1400 nm range compared to *Bryoria* (Fig. 2).

Desiccation did not significantly alter the shape of the reflectance spectra, but the difference between the two species diminished after drying (Fig. 2). Drying increased the reflectance between 420 and 1400 nm in the melanic lichen and decreased it in the usnic species. The PAR reflectance of *Bryoria* was significantly higher in dry thalli compared to wet thalli, as evidenced by non-overlapping 95% confidence intervals. In contrast, the visible reflectance between dry and wet in *Alectoria* did not significantly differ (Fig. 2). Although the green peak was reduced after drying, it remained distinct in dry *Alectoria*, but was barely visible in the dry *Bryoria* (Fig. 2).

At approximately 1400 nm, the reflectance spectra of both species abruptly declined and converged, showing no more significant differences between the species up to the highest measured wavelengths at 2500 nm (Fig. 2; insert). However, hydration reduced the reflectance in both species between 1400 and 2500 nm.

### Light response curves of ETR_App_

**Fig. 3:**
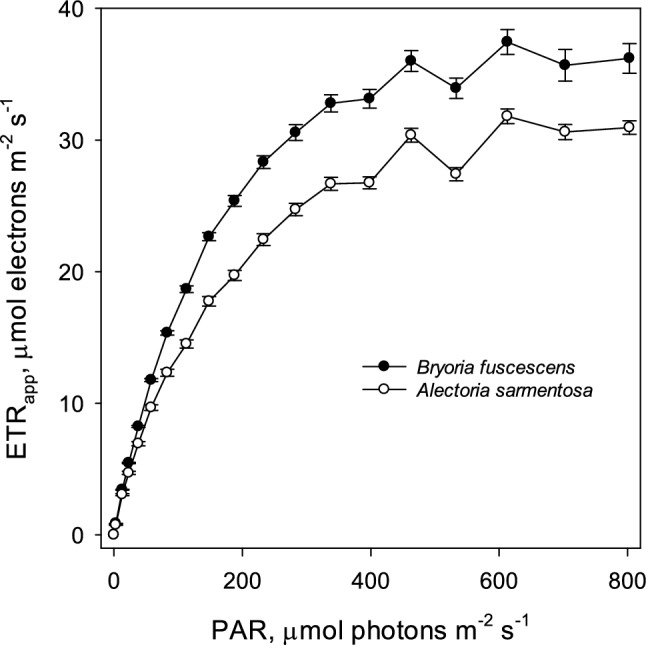
Light saturation curves of mean apparent electron transport rate (ETR_App_) in spring for the usnic *Alectoria sarmentosa* (open symbols) and the darkly melanic *Bryoria fuscescens* (black symbols) measured with red actinic light. Error bars show standard error (*n* = 12)

*Bryoria* exhibited significantly higher ETR_App_ compared to *Alectoria*, with the gap widening as light intensity rose (Fig. 3). Despite this difference, the light response curves for both species had a similar shape, showing light saturation at 500–600 μmol photons m^−2^ s^−1^. The relative difference between the species remained fairly constant with increasing light intensity.

### Fast recovery after high-light exposures of hydrated thalli

**Fig. 4:**
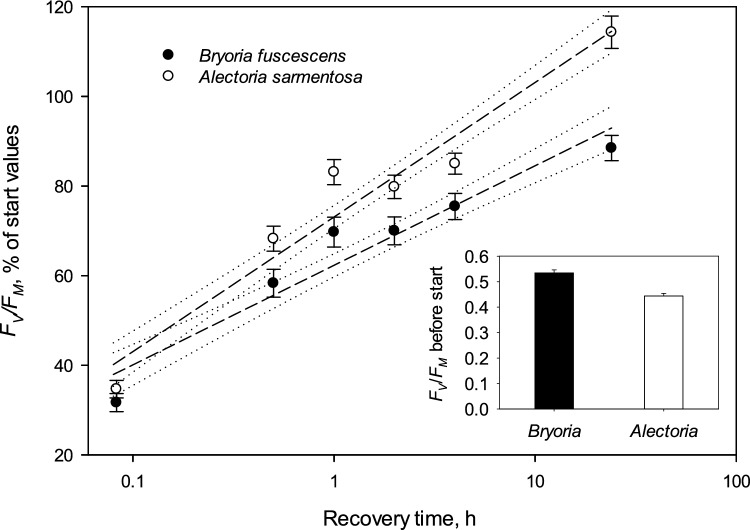
The mean kinetics of recovery from photoinhibition after a 4-h exposure of 750 μmol photons m^−2^ s^−1^ for hydrated thalli of the usnic *Alectoria sarmentosa* (open symbols; *R*^2^_adj_ = 0.840; *P* <0.001) and the darkly melanic *Bryoria fuscescens* (black symbols; *R*^2^_adj_ = 0.752; *P* <0.001). *F*_V_*/F*_M_ is expressed as percent of the pre-start values of dark-adapted specimens. The inset shows the mean *F*_V_*/F*_M_ of both species at start. Error bars show standard error (*n* = 12). The regression line with corresponding 95% confidence interval (dotted lines) is given for each species

**Fig. 5:**
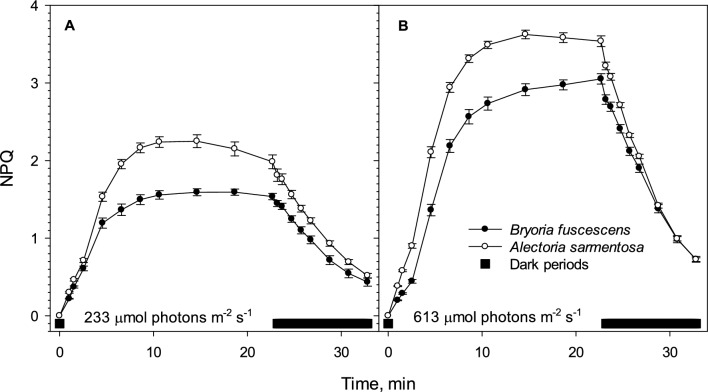
The mean kinetics of non-photochemical quenching (NPQ) in dark-adapted intact thalli of the usnic *Alectoria sarmentosa* (open symbols) and the darkly melanic *Bryoria fuscescens* (black symbols). Thalli were exposed to 233 (**A**) and 613 (**B**) μmol photons m^−2^ s^−1^, respectively, for 23 min followed by a 10 min dark period (shown by the thick horizontal black bar). Error bars show mean ± SE when larger than symbol size (*n* = 12)

The maximum quantum yield of PSII (*F*_*V*_*/F*_*M*_) after 24 h of low-light pre-treatment was low in both *Alectoria* (0.444±0.011; mean ± SE) and *Bryoria* (0.535±0.012; Fig. 5, inset). A 4-h high-light exposure at 750 μmol photons m^−2^ s^−1^ reduced *F*_*V*_*/F*_*M*_ to one-third of initial values in both species. However, photoinhibition recovered within 24 h under low light (Fig. 4). Recovery kinetics showed a rather linear increase along the log-transformed time scale, with highly significant linear regression lines (*P* < 0.001) for both *Bryoria* (*R*^2^_adj_ = 0.752) and *Alectoria R*^2^_adj_ = 0.840). Notably, *Alectoria* recovered much faster than *Bryoria*, reaching initial values within 10 h of recovery (Fig. 4). This was evidenced by non-overlapping 95% confidence intervals after 30 min of recovery (Fig. 4). Consequently, the difference in *F*_*V*_*/F*_*M*_ between the species diminished significantly after 24 h recovery (*Alectoria*: 0.505±0.015; *Bryoria*: 0.471±0.009; mean ± SE).

### Non-photochemical quenching

NPQ increased with light intensity but responded more strongly and quickly to sudden light exposure in *Alectoria* compared to *Bryoria* (Fig. 5). After an initial linear increase in NPQ lasting approximately 2.5 min, NPQ accelerated for the next 2 min in both hair lichens. The increase then slowed, peaking after 15 min in *Alectoria* while NPQ continued to rise at a slower rate in *Bryoria*, peaking at 19 min under 233 μmol photons m^−2^ s^−1^ light exposure (Fig. 5A), or continuing to increase until end of the 23-min exposure at 613 μmol photons m^−2^ s^−1^ light exposure (Fig. 5B).

When the light was switched off, NPQ rapidly relaxed, with the fastest rate observed after the highest light exposure. Relaxation was slightly faster in *Alectoria* than in *Bryoria* (Fig. 5), but both species reached the same NPQ-level at the end of the 10-min dark exposure.

### Chlorophylls

Despite their similar morphology, the darkly pigmented *Bryoria* had a 1.8 times higher total Chl concentration (0.698±0.075 mg g^−1^; mean ± SE) than the sympatric, but pale *Alectoria* (*P* = 0.009). Additionally, *Bryoria* had 1.5 times higher Chl *a/b*-ratio (4.87±0.26) than *Alectoria* (*P* = 0.002; Table 1).

**Table 1:** Total chlorophyll concentration and chlorophyll *a/b*-ratio in the hair lichens *Alectoria sarmentosa* and *Bryoria fuscescens*

	*Alectoria sarmentosa*	*Bryoria fuscescens*	*t* value	*P* value
Total Chl (mg g^−1^)	0.397 ± 0.028	0.698 ± 0.075	− 3.78	0.009
Chl *a/b*-ratio	3.31 ± 0.05	4.87 ± 0.26	− 5.75	0.002

Mean values ± standard error (*n* = 6) are given.

## Discussion

Lichens are remarkable successful photosynthetic organisms, thriving in some of the most sun-exposed and extreme habitats on Earth (Kappen 1973). They can survive extended exposure to outer space (Sancho et al. 2007; Onofri et al. 2012) and photosynthesize under simulated Martian conditions (de Vera et al. 2010; de Vera 2012), indicating their possession of highly effective photoprotective mechanisms. Lichens utilize a variety of sun-screening fungal pigments, such as parietin (Solhaug and Gauslaa 1996), atranorin (Solhaug et al. 2010), usnic acid (McEvoy et al. 2006; 2007), vulpinic acid (Phinney et al. 2019), and melanin (Gauslaa and Solhaug 2001). Comparing the efficiency of screening mechanisms is complex due to the different ways in which reflecting pigments like usnic acid and absorbing pigments like melanin handle solar radiation (Gauslaa and Goward 2023). However, our study provides valuable insight into the efficiency of these cortical pigments through the photobiological responses of sympatric usnic and melanic hair lichens.

The usnic *Alectoria* displayed a significantly stronger green reflectance peak than *Bryoria*, indicating greater exposure of its Chl to ambient light outside the screening lichen cortices in *Alectoria*. This occurs despite *Bryoria* having a higher total Chl concentration than *Alectoria*. However, when Chl concentrations were adjusted to Chl content per thallus area, using specific thallus mass data provided by Esseen et al. (2015) for hair lichens, the contrast between species was markedly reduced. In the melanic species, Chl was less visible through a hydrated cortex, as evidenced by its considerably weaker green reflectance. Upon desiccation, *Bryoria*’s red reflectance increased more than its green reflectance, causing the faint green tinge to disappear, whereas the green reflectance peak remained distinct in *Alectoria*. This pattern is also observed in other pairs of melanic and usnic hair lichen species (Gauslaa 1984). The higher visibility of Chl in the usnic species suggests greater cortical transmittance, which likely contributes to the increased susceptibility to high light in desiccated usnic hair lichens compared to melanic ones (Färber et al. 2014).

During high-light exposures while hydrated, *Alectoria* exhibited significantly higher and more rapidly induced NPQ, as well as faster relaxation in darkness, compared to its melanic counterpart. This aligns with the recent findings showing more flexible NPQ responses to high light exposures in usnic versus melanic species of sympatric mat-forming alpine lichens (Solhaug et al. 2024). Both usnic and melanic populations of these thick mat-forming lichens, as well as the thin hair lichens in this study, had acclimated to similar external light conditions by forming species-specific cortical pigments. We assume that hydrated lichen species exposed to the same external light condition require a similar ability to handle ambient light, but that their set of tools to manage excess excitation energy differs. Given NPQ’s flexibility and rapid response, our data suggest that insufficient fungal screening could be compensated by the measured enhanced NPQ observed in *Alectoria* photobionts.

NPQ in hair lichens exposed to light tended to decrease over time, especially in the usnic species and at lower light level. This decline may occur, because some time is needed for full activation of photosynthesis, which, once activated, can handle more light, reducing the need for NPQ to mitigate oxidative stress. The stronger decline in NPQ in mat-forming lichens (Solhaug et al. 2024) compared to hair lichens suggests that thin hair lichens need shorter time for full photosynthetic activation.

*Alectoria* recovered more quickly than *Bryoria* from photoinhibition experienced after a 4-h high-light treatment in the hydrated state (Fig. 4), a response opposite to that observed in desiccated thalli (Färber et al. 2014). The fast recovery of *Alectoria* at low light after photoinhibition is not entirely understood. Its initial *F*_*V*_*/F*_*M*_ was lower than that of *Bryoria*, likely due to the exceptionally sunny and dry weather before sampling, which may have affected *Alectoria* more. Thus, the post-stress 24-h relaxation at low light could also have allowed some recovery from residual photoinhibition experienced in the field before the artificial high-light treatment. In seasonal climates, normal acclimation involves a significant reduction in *F*_*V*_*/F*_*M*_ during periods when photosynthesis is constrained by factors such as low temperature or drought, which limit the ability to manage excess light (e.g., Vrábliková et al. 2006; Veres et al. 2022). Vernal photoinhibition observed in indigenous lichens from pristine, unpolluted forest hardly indicates impaired viability.

The two sympatric hair lichens exhibited light saturation at relatively high light levels, aligning with the open and sun-exposed canopies from which they were sampled (Fig. 1). A higher ETR_App_ in one species suggests a greater degree of cortical screening, assuming that the relative increase is similar across various irradiance levels. Conversely, a higher ETR_App_ observed only at high irradiance indicates enhanced photosynthetic capacity. The combination of higher ETR_App_ in *Bryoria* and a similar relative rise in ETR_App_ with increasing light for both species suggests more effective fungal screening in *Bryoria* compared to *Alectoria*. This is corroborated by the strong green reflectance peak in *Alectoria* (Fig. 2), suggesting that more light penetrates though the cortex and reaches the photobiont layer beneath the usnic cortex compared to the melanic cortex. Finally, the more pronounced drying-induced relative reduction of the green reflectance in *Bryoria* than in *Alectoria* (Fig. 2) aligns with the reported strong high-light tolerance in desiccated thalli of melanic hair lichens (Färber et al. 2014).

In conclusion, melanin serves as a more efficient sunscreen than usnic acid in thin hair lichens. The robust screening by dry melanic cortices enables *Bryoria* to thrive in the upper tree canopies of drier climates during extended dry and sunny periods when NPQ cannot operate. In contrast, the usnic lichen *Alectoria*, with less-effective solar radiation screening, exhibits the highest and most rapidly induced NPQ when hydrated. This supports the hypothesis that the fungal pigment usnic acid alone is inadequate for preventing photoinhibitory damage in sun-exposed hair lichens. *Alectoria’*s preference for lower humid canopy layers exposed to intermittent sunflecks is likely facilitated by its adaptable NPQ, optimizing photosynthesis during hydration periods under fluctuating light conditions. *Alectoria*’s success in well-lit upper canopies of rainforest (Benson and Coxson 2002; Antoine and McCune 2004) hinges on frequent hydration, which not only enables NPQ but also repairs photoinhibitory damage that would otherwise accumulate in drier climates. While the mycobiont offers primary photoprotection in dry lichens, the NPQ of the photobiont complements the photoprotective role of fungal pigments in hydrated lichens.

## Data Availability

Original excel files for the data shown in Figures and Tables are available on request.

## Abbreviations

ETR: Electron transport rate
ETR_App_: Apparent ETR
NPQ: Non-photochemical quenching
PAR: Photosynthetic active light

## References

[CR1] Ahti T (1977) Lichens of the boreal coniferous zone. In: Seaward MRD (ed) Lichen ecology. Academic Press, London, pp 145–181

[CR2] Antoine ME, McCune B (2004) Contrasting fundamental and realized ecological niches with epiphytic lichen transplants in an old-growth *Pseudotsuga* forest. Bryologist 107(2):163–172. 10.1639/0007-2745(2004)107[0163:CFAREN]2.0.CO;2

[CR3] Baker NR (2008) Chlorophyll fluorescence: a probe of photosynthesis in vivo. Ann Rev Plant Biol 59:89–113. 10.1146/annurev.arplant.59.032607.092759 18444897 10.1146/annurev.arplant.59.032607.092759

[CR4] Beckett RP, Minibayeva F, Solhaug KA, Roach T (2021) Photoprotection in lichens: adaptations of photobionts to high light. Lichenologist 53:21–33. 10.1017/S0024282920000535

[CR5] Beilinson Y, Rassabina A, Lunev I, Faizullin D, Greenbaum A, Salnikov V, Zuev Y, Minibayeva F, Feldman Y (2022) The dielectric response of hydrated water as a structural signature of nanoconfined lichen melanins. Phys Chem Chem Phys 24:22624–22633. 10.1039/D2CP01383E 36102934 10.1039/d2cp01383e

[CR6] Benson S, Coxson DS (2002) Lichen colonization and gap structure in wet-temperate rainforests of northern interior British Columbia. Bryologist 105:673–692. 10.1639/0007-2745(2002)105[0673:LCAGSI]2.0.CO;2

[CR7] Boudreault C, Drapeau P, Bouchard M, St-Laurent M-H, Imbeau L, Bergeron Y (2015) Contrasting responses of epiphytic and terricolous lichens to variations in forest characteristics in northern boreal ecosystems. Can J for Res 45:595–606. 10.1139/cjfr-2013-0529

[CR8] Brodo IM, Hawksworth DL (1977) *Alectoria* and allied genera in North America. Opera Botanica 42:1–164

[CR9] Campbell J, Coxson DS (2001) Canopy microclimate and arboreal lichen loading in subalpine spruce-fir forest. Can J Bot 79:537–555. 10.1139/b01-025

[CR10] Coxson DS, Coyle M (2003) Niche partitioning and photosynthetic response of alectorioid lichens from subalpine spruce-fir forest in north-central British Columbia, Canada: the role of canopy microclimate gradients. Lichenologist 35:157–175. 10.1016/S0024-2829(03)00018-5

[CR11] Coxson DS, Stevenson SK (2007) Growth rate responses of *Lobaria pulmonaria* to canopy structure in even-aged and old-growth cedar-hemlock forests of central-interior British Columbia, Canada. For Ecol Manage 24:5–16. 10.1016/j.foreco.2007.01.031

[CR12] de Vera J-P (2012) Lichens as survivors in space and on Mars. Fungal Ecol 5:472–479. 10.1016/j.funeco.2012.01.008

[CR13] de Vera JP, Möhlmann D, Butina F, Lorek A, Wernecke R, Ott S (2010) Survival potential and photosynthetic activity of lichens under Mars-like conditions: a laboratory study. Astrobiology 10:215–227. 10.1089/ast.2009.0362 20402583 10.1089/ast.2009.0362

[CR14] Demmig-Adams B, Koh S-C, Cohu CM, Muller O, Stewart JJ, Adams WW (2014) Non-photochemical fluorescence quenching in contrasting plant species and environments. In: Demmig-Adams B, Garab G, Adams W, Govindjee, (eds) Non-photochemical quenching and energy dissipation in plants algae and cyanobacteria. Springer, Netherlands, Dordrecht

[CR15] Edwards RY, Soos J, Ritcey RW (1960) Quantitative observations on epidendric lichens used as food by caribou. Ecology 41:425–431

[CR16] Eriksson A, Gauslaa Y, Palmqvist K, Ekström M, Esseen PA (2018) Morphology drives water storage traits in the globally widespread lichen genus *Usnea*. Fungal Ecol 35:51–61. 10.1016/j.funeco.2018.06.007

[CR17] Esseen PA, Coxson DS (2024) Microclimate drives growth of hair lichens in boreal forest canopies after partial cutting. For Ecol Manage 572:122319. 10.1016/j.foreco.2024.122319

[CR18] Esseen P-A, Renhorn KE, Pettersson RB (1996) Epiphytic lichen biomass in managed and old-growth boreal forests: effect of branch quality. Ecol Appl 6:228–238. 10.2307/2269566

[CR19] Esseen P-A, Olsson T, Coxson D, Gauslaa Y (2015) Morphology influences water storage in hair lichens from boreal forest canopies. Fungal Ecol 18:26–35. 10.1016/j.funeco.2015.07.008

[CR20] Esseen P-A, Rönnqvist M, Gauslaa Y, Coxson DS (2017) Externally held water – a key factor for hair lichens in boreal forest canopies. Fungal Ecol 30:29–38. 10.1016/j.funeco.2017.08.003

[CR21] Färber L, Solhaug KA, Esseen P-A, Bilger W, Gauslaa Y (2014) Sunscreening fungal pigments influence the vertical gradient of pendulous lichens in boreal forest canopies. Ecology 95:1464–1471. 10.1890/13-2319.1 25039211 10.1890/13-2319.1

[CR22] Gauslaa Y (1984) Heat resistance and energy budget in different Scandinavian plants. Ecography 7:1–78. 10.1111/j.1600-0587.1984.tb01098.x

[CR23] Gauslaa Y, Goward T (2023) Sunscreening pigments shape the horizontal distribution of pendent hair lichens in the lower canopy of unmanaged coniferous forests. Lichenologist 55:81–89. 10.1017/S0024282923000075

[CR24] Gauslaa Y, Solhaug KA (2001) Fungal melanins as a sun screen for symbiotic green algae in the lichen *Lobaria pulmonaria*. Oecologia 126:462–471. 10.1007/s004420000541 28547230 10.1007/s004420000541

[CR25] Goss R, Lepetit B (2015) Biodiversity of NPQ. J Plant Physiol 172:13–32. 10.1016/j.jplph.2014.03.004 24854581 10.1016/j.jplph.2014.03.004

[CR26] Goward T (1998) Observations on the ecology of the lichen genus *Bryoria* in high elevation conifer forests. Can Field Nat 112:496–501

[CR27] Goward T, Gauslaa Y, Björk CR, Woods D, Wright KG (2022) Stand openness predicts hair lichen (*Bryoria*) abundance in the lower canopy, with implications for the conservation of Canada’s critically imperiled Deep-Snow Mountain Caribou (*Rangifer tarandus caribou*). For Ecol Manage 520:120416. 10.1016/j.foreco.2022.120416

[CR28] Goward T, Coxson D, Gauslaa Y (2024) The Manna effect – a review of factors influencing hair lichen abundance for Canada’s endangered Deep-Snow Mountain Caribou (*Rangifer arcticus montanus*). Lichenologist 56:121–135. 10.1017/S0024282924000161

[CR29] Heggberget TM, Gaare E, Ball JP (2002) Reindeer *(Rangifer tarandus)* and climate change: importance of winter forage. Rangifer 22:13–31. 10.7557/2.22.1.388

[CR30] Honegger R (2003) The impact of different long-term storage conditions on the viability of lichen-forming ascomycetes and their green algal photobiont, *Trebouxia* spp. Plant Biol 5:324–330. 10.1055/s-2003-40794

[CR31] Horstkotte T, Moen J, Lämås T, Helle T (2011) The legacy of logging—estimating arboreal lichen occurrence in a boreal multiple-use landscape on a two century scale. PLoS ONE 6(12):e28779. 10.1371/journal.pone.0028779 22194912 10.1371/journal.pone.0028779 PMC3241682

[CR32] Jung H-S, Niyogi KK (2006) Molecular analysis of photoprotection of photosynthesis. In: Demmig-Adams B, Adams WW, Mattoo AK (eds) Photoprotection, photoinhibition gene regulation, and environment. Springer, Netherlands, Dordrecht, pp 127–143

[CR33] Kappen L (1973) Response to extreme environments. In: Ahmadjian V (ed) The lichens. Academic Press, New York, pp 311–380

[CR34] Knops JMH, Nash TH III, Schlesinger WH (1996) The influence of epiphytic lichens on the nutrient cycling of an oak woodland. Ecol Monographs 66:159–179. 10.2307/2963473

[CR35] Lange OL, Green TGA, Heber U (2001) Hydration-dependent photosynthetic production of lichens: what do laboratory studies tell us about field performance? J Exp Bot 52:2033–2042. 10.1093/jexbot/52.363.2033 11559739 10.1093/jexbot/52.363.2033

[CR36] McEvoy M, Nybakken L, Solhaug KA, Gauslaa Y (2006) UV triggers the synthesis of the widely distributed secondary compound usnic acid. Mycol Progress 5:221–229. 10.1007/s11557-006-0514-9

[CR37] McEvoy M, Solhaug KA, Gauslaa Y (2007) Solar radiation screening in usnic acid-containing cortices of the lichen *Nephroma arcticum*. Symbiosis 43:143–150

[CR38] Mkhize KGW, Minibayeva F, Beckett RP (2022) Adaptions of photosynthesis in sun and shade in populations of some Afromontane lichens. Lichenologist 54:319–329. 10.1017/S0024282922000214

[CR39] Moen A (1999) National atlas of Norway: Vegetation. Norwegian Mapping Authority, Hønefoss

[CR40] Onofri S, de la Torre R, de Vera J-P, Ott S, Zucconi L, Selbmann L, Scalzi G, Venkateswaran KJ, Rabbow E, Sanchez Inigo FJ, Horneck G (2012) Survival of rock-colonizing organisms after 1.5 years in outer space. Astrobiology 12:508–5016. 10.1089/ast.2011.0736 22680696 10.1089/ast.2011.0736

[CR41] Pettersson RB, Ball JP, Renhorn KE, Esseen P-A, Sjöberg K (1995) Invertebrate communities in boreal forest canopies as influenced by forestry and lichens with implications for passerine birds. Biol Conservation 74:57–63. 10.1016/0006-3207(95)00015-V

[CR42] Phinney NH, Gauslaa Y, Solhaug KA (2019) Why chartreuse? The pigment vulpinic acid screens blue light in the lichen *Letharia vulpina*. Planta 249:709–718. 10.1007/s00425-018-3034-3 30374913 10.1007/s00425-018-3034-3

[CR43] Phinney NH, Gauslaa Y, Palmqvist K, Esseen P-A (2021) Macroclimate drives growth of hair lichens in boreal forest canopies. J Ecol 109:478–490. 10.1111/1365-2745.13522

[CR44] Price K, Hochachka G (2001) Epiphytic lichen abundance: effects of stand age and composition in coastal British Columbia. Ecol Appl 11:904–913. 10.1890/1051-0761(2001)011[0904:ELAEOS]2.0.CO;2

[CR45] Pypker TG, Unsworth MH, Van Stan JT, Bond BJ (2017) The absorption and evaporation of water vapor by epiphytes in an old-growth Douglas-fir forest during the seasonal summer dry season: Implications for the canopy energy budget. Ecohydrology 10(3):e1801. 10.1002/eco.1801

[CR46] Rominger EM, Oldemeyer JL (1990) Early-winter diet of woodland caribou in relation to snow accumulation, Selkirk Mountains, British Columbia, Canada. Can J Zool 68:2691–2694. 10.1139/z90-372

[CR47] Rominger EM, Robbins CT, Evans MA (1996) Winter foraging ecology of woodland caribou in northeastern Washington. J Wildl Manage 60:719–728. 10.2307/3802370

[CR48] Sancho LG, De la Torre R, Horneck G, Ascaso C, De los Rios A, Pintado A, Wierzchos J, Schuster M (2007) Lichens survive in space: Results from the 2005 LICHENS experiment. Astrobiology 7:443–454. 10.1089/ast.2006.00466 17630840 10.1089/ast.2006.0046

[CR49] Schreiber U, Schliwa U, Bilger W (1986) Continuous recording of photochemical and non-photochemical chlorophyll fluorescence quenching with a new type of modulation fluorometer. Photosynth Res 10:51–62. 10.1007/BF00024185 24435276 10.1007/BF00024185

[CR50] Solhaug KA (2018) Low-light recovery effects on assessment of photoinhibition with chlorophyll fluorescence in lichens. Lichenologist 50:139–145. 10.1017/S0024282917000640

[CR51] Solhaug KA, Gauslaa Y (1996) Parietin, a photoprotective secondary product of the lichen *Xanthoria parietina*. Oecologia 108:412–418. 10.1007/BF00333715 28307855 10.1007/BF00333715

[CR52] Solhaug KA, Gauslaa Y (2025) Quantifying chlorophylls in melanic lichens: the necessity of separating the absorbance of melanin and chlorophyll. Photosynth Res 163:17. 10.1007/s11120-025-01141-w 39937322 10.1007/s11120-025-01141-w PMC11821740

[CR53] Solhaug KA, Larsson P, Gauslaa Y (2010) Light screening in lichen cortices can be quantified by chlorophyll fluorescence techniques for both reflecting and absorbing pigments. Planta 231:1003–1011. 10.1007/s00425-010-1103-3 20135325 10.1007/s00425-010-1103-3

[CR54] Solhaug KA, Eiterjord G, Løken MH, Gauslaa Y (2024) Non-photochemical quenching may contribute to the dominance of the pale mat-forming lichen *Cladonia stellaris* over the sympatric melanic *Cetraria islandica*. Oecologia 204:187–198. 10.1007/s00442-023-05498-4 38233688 10.1007/s00442-023-05498-4 PMC10830725

[CR55] Stevenson SK (2001) Arboreal lichens. In: Jull MJ, Stevenson SK (eds) The Lucille Mountain Study: 8-year results of a silvicultural systems trial in the Engelmann spruce-subalpine fir Zone. British Columbia Ministry of Forests. https://www.for.gov.bc.ca/hfd/pubs/docs/wp/wp59.htm (accessed 29 October 2023). BC Ministry of Forests Working Paper 59. Victoria, British Columbia, pp 60-65

[CR56] Veres K, Sinigla M, Szabó K, Varga N, Farkas E (2022) The long-term effect of removing the UV-protectant usnic acid from the thalli of the lichen *Cladonia foliacea*. Mycol Prog 21(9):83. 10.1007/s11557-022-01831-y 36065212 10.1007/s11557-022-01831-y PMC9433529

[CR57] Vrábliková H, McEvoy M, Solhaug KA, Barták M, Gauslaa Y (2006) Annual variation in photo acclimation and photoprotection of the photobiont in the foliose lichen *Xanthoria parietina*. J Photochem Photobiol b: Biology 83:151–162. 10.1016/j.jphotobiol.2005.12.019 16481192 10.1016/j.jphotobiol.2005.12.019

[CR58] Way DA, Pearcy RW (2012) Sunflecks in trees and forests: from photosynthetic physiology to global change biology. Tree Physiol 32:1066–1081. 10.1093/treephys/tps064 22887371 10.1093/treephys/tps064

[CR59] Wellburn AR (1994) The spectral determination of chlorophyll *a* and *b*, as well as total carotenoids, using various solvents with spectrophotometers of different resolution. J Plant Physiol 144:307–313. 10.1016/S0176-1617(11)81192-2

